# Genome-wide association study of the antibody response to *Corynebacterium pseudotuberculosis* in sheep

**DOI:** 10.5194/aab-68-109-2025

**Published:** 2025-02-11

**Authors:** Jitka Kyselová, Ladislav Tichý, Jiřina Marková, Artur Gurgul, Zuzana Sztankóová, Karel Vališ, Soňa Šlosárková, Kateřina Kavanová, Monika Beinhauerová, Tomasz Szmatola

**Affiliations:** 1 Department of Genetics and Breeding of Farm Animals, Institute of Animal Science, 104 00 Prague, Czech Republic; 2 Department of Microbiology and Antimicrobial Resistance, Veterinary Research Institute, 621 00 Brno, Czech Republic; 3 Centre for Experimental and Innovative Medicine, University of Agriculture in Krakow, 30-248 Kraków, Poland; 4 Department of Infectious Diseases and Preventive Medicine, Veterinary Research Institute, 621 00 Brno, Czech Republic; 5 Department of Animal Molecular Biology, National Research Institute of Animal Production, 32-083 Balice, Poland

## Abstract

Caseous lymphadenitis (CLA) is a chronic and highly contagious disease that is widespread among sheep and goats and adversely affects animal welfare and production. This study aimed to reveal the host genomic influences on *Corynebacterium pseudotuberculosis* antibody levels as indicators of disease susceptibility or resistance, thereby potentially identifying genetic markers associated with these traits. Blood samples were collected from 321 sheep from four large Czech sheep herds. Animals of the Suffolk breed, ranging in age from 2 to 8 years, were sampled regularly once a year for 4 years. The sheep were classified into healthy and diseased groups based on two different commercial enzyme-linked immunosorbent assay (ELISA) serological antigen tests. Genomic DNA was genotyped with the GeneSeek^®^ Genomic Profiler™ Ovine 50 K, and 41 301 markers were used for the genome-wide association analysis (GWAS). A case–control GWAS including 143 seropositive and 178 seronegative sheep was conducted to assess the relationship between the sheep genome and the antibody response to a phospholipase D antigen of *C. pseudotuberculosis* using GCTA software. The study revealed two suggestive SNPs (single-nucleotide polymorphisms) on chromosomes 11 and 20, with the most significant SNP in the first exon of the *TRIM16* (tripartite-motif-containing 16) gene. By analysing genomic alterations and their context between positive and negative animals, including a gene ontology (GO) analysis of genes within ± 500 kb regions of the suggestive SNPs, we identified genes and immune-related processes and pathways potentially influencing CLA susceptibility. These include antigen processing and presentation of exogenous peptides via major histocompatibility complex (MHC) class I and II, Th17 mucosal cell differentiation, cellular autophagy, and phagosome-related mechanisms. This research provides insights into the genetic basis of the antibody response to *C. pseudotuberculosis*, identifying suggestive associations and underlying biological mechanisms that could guide future breeding and genetic strategies for improving resistance to CLA in sheep.

## 1 Introduction

Caseous lymphadenitis (CLA), a chronic and highly contagious disease in sheep and goats, poses significant challenges to animal health and production. It is primarily caused by *C. pseudotuberculosis* (*Cps*), which is a facultative anaerobic, Gram-positive, intracellular, pleomorphic bacterium that leads to the development of pyogranulomas or abscesses in lymphatic tissues, both superficially and in internal organs (Pepin and Paton, 2011). Internal abscesses often remain asymptomatic until advanced stages, making early detection challenging. The incubation period of CLA ranges from 2 to 6 months, during which clinical signs may not be evident. Affected sheep may exhibit intermittent fever, reduced feed intake, and lethargy, which contribute to poor body condition and weight loss. Antibodies against *Cps* typically exceed diagnostic thresholds within the first few weeks following infection. Direct transmission routes, such as physical contact and environmental contamination, contribute to its rapid spread within herds. Moreover, *Cps* has zoonotic potential, with the first documented case of human infection reported in 1966. Although rare, transmission to humans can occur, posing an occupational hazard for individuals in close contact with infected animals (Bastos et al., 2012; Lopez et al., 1966). Once introduced into a flock, CLA becomes difficult to control; sheep often become lifelong carriers, and spontaneous recovery is rare (Leite-Browning, 2019; Fontaine and Baird, 2008; Farhan et al., 2023; Pepin and Paton, 2011). The poor response of CLA to antibiotic treatment further complicates the situation, making its complete eradication problematic, expensive, and time-consuming (Dominguez et al., 2021). Effective strategies are urgently needed to manage and control CLA, highlighting the importance of research in this field.

*Cps's* virulence is mainly attributed to two primary factors: a waxy, mycolic acid-rich cell wall and a potent exotoxin-specific phospholipase D (PLD). PLD facilitates the spread of bacteria within the host and triggers immunoglobulin (IgG) antibody responses in infected animals (Zamprogna et al., 2021). Several studies on the serological monitoring of CLA have demonstrated the effectiveness of antibody detection assays, which play a crucial role in identifying infected animals and managing the disease (Moura et al., 2020; Costa et al., 2020; Bettini et al., 2022). Research by Kuria and Holstad (1989) emphasized the correlation between haemolysis-inhibition tests and enzyme-linked immunosorbent assays (ELISAs) for detecting *Cps* antibodies, underscoring the need for accurate serological testing. Further studies identified immunodominant antigens in *Cps*, enhancing our understanding of antibody responses in infected animals (Muckle et al., 1992; Reboucas et al., 2020; Galvao et al., 2017; Santos, 2016). An indirect ELISA was developed using antigens secreted from the *Cps* T1 strain (Reboucas et al., 2013). The assay reached a sensitivity of 89 % and a specificity of 99 %, demonstrating its effectiveness in serodiagnosis in sheep. ELISA is currently the most common test used in live animals, providing a reliable disease control tool.

Using molecular information in breeding has significantly improved livestock production by enhancing traits of economic interest. Recent advancements in genomics and computational techniques have allowed for a better genetic understanding of farm animals and the identification of novel mutations and SNPs (single-nucleotide polymorphisms) associated with various traits (Barbosa et al., 2023). Case–control genome-wide association studies offer distinct advantages for studying disease susceptibility, particularly for infectious diseases like CLA. This approach compares genetic variations between affected and unaffected animals, making it a powerful tool for identifying genetic markers and candidate genes associated with disease resistance or susceptibility in sheep populations. Previous studies have successfully applied case–control genome-wide association analysis (GWAS) to identify genetic factors underlying various infectious diseases in sheep and goats, including lentiviral infections, paratuberculosis, and pneumonia (Cecchi et al., 2019; Martin et al., 2021; Moioli et al., 2016).

A study by Moioli et al. (2016) performed a GWAS of paratuberculosis susceptibility based on ELISA phenotypes and identified associated genes. Recent genomic and proteomic analyses have begun to identify genetic susceptibility markers for CLA in goats (Minozzi et al., 2017; Fu et al., 2020; Fu et al., 2021). However, a considerable knowledge gap exists in understanding the complex interaction between the sheep genome and the *Cps* pathogen. The biological mechanisms underlying individual resistance to CLA are still unknown, and identifying genome markers associated with resistance or susceptibility may help develop breeding strategies, better diagnostic tools, and alternative treatments (McRae et al., 2018).

In this study, we hypothesized that long-term serologically negative, healthy animals exhibit greater resistance or tolerance, whereas serologically positive, diseased animals are more sensitive or less tolerant to *Cps *infection. The aim of the study was to explore the genetic basis of antibody production against *Cps* by utilizing SNP genotypes spanning the entire ovine genome and serological ELISA data collected over 4 years from investigated sheep farms. Identifying SNPs, overlapping genes, and enriched functional ontological terms associated with antibody response and CLA susceptibility is critical, given the significant health risks this pathogen poses to small ruminants and its potential zoonotic implications for humans.

## 2 Materials and methods

### 2.1 Animals and sampling procedure

Initially, 30 sheep farms in the Czech Republic were evaluated for the occurrence of CLA by testing serum antibodies against *Cps *using ELISA. Subsequently, entire herds from several farms with confirmed *Cps* presence underwent regular ELISA testing annually for 4 years. Purebred Suffolk sheep selected for the association study originated from four farms (i.e. four different herds); the animals were maintained under comparable housing conditions and nutritional regimens, were in daily and night contact, and met the conditions of free pathogen spreading. The sheep were managed in a mixed-housing system, which varied according to climatic conditions. From March to November, they grazed on pasture, whereas they were housed in indoor barns during the winter and fed hay or haylage ad libitum, with minimal grain meal supplementation. Salt and mineral licks were available year-round. Specific data on metabolizable energy intake and fibre content were not available for the tested herds. The physical condition of the animals, clinical signs of disease, and deworming strategies were recorded. For downstream laboratory analyses, peripheral blood samples from 321 suitable sheep aged between 2 and 8 years were collected via the jugular vein. Blood was divided into two sterile 3 mL centrifugation tubes each. Tubes containing 25 µ L of 0.5 µ M ethylenediaminetetraacetic acid (EDTA) anticoagulant were immediately cooled and stored at - 20 °C until DNA extraction. Blood samples for serum antibody testing were collected in tubes and kept at room temperature for 24 h to allow clotting. The samples were then centrifuged using an Eppendorf Centrifuge 5810R at 2500 ×g for 2 min to obtain high-quality serum, which was subsequently used for ELISA.

### 2.2 DNA extraction and SNP genotyping of animals

Genomic DNA was isolated from 200 µ L of cryopreserved whole blood from ewes using the NucleoMag^®^ Blood kit (MACHEREY-NAGEL, Düren, Germany) on an epMotion^®^ 5073 m (Eppendorf AG, Hamburg, Germany) automatic pipetting instrument, according to the vendor's recommended protocol. The DNA quantity and quality were assessed by a NanoDrop 1000 spectrophotometer and Qubit 4 fluorometer (Thermo Fisher Scientific, Waltham, MA, USA), respectively. All samples met the 260/280 ratio criterion of 2.0–2.2 and the 260/230 criterion of 1.8–2.0, respectively. The average yield achieved was 75.2 ng µ L^−1^ of DNA. Purified genomic DNA was genotyped with the GeneSeek^®^ Genomic Profiler™ (GGP) Ovine 50 K chip at the laboratory of NEOGEN^®^ Genomics Europe Ltd. (Ayr, UK). Quality control of the genotypes was performed using PLINK v1.9 software (Purcell et al., 2007; Chang et al., 2015). Samples with an individual genotyping call rate of less than 97 % were applied for filtering. We removed SNPs that had an unknown genomic location, those that mapped onto sex chromosomes, those that had a minor allele frequency of less than 0.05, those with a genotype failure > 20 %, and those that departed from the Hardy–Weinberg equilibrium with a p value < $1\times 10^{-7}$ . By doing so, we retained 41 301 SNP markers for the association analysis (the number of markers utilized on the GGP Ovine 50K chip was 51 867), ensuring the most reliable results possible. The basic population characteristics, such as the overall level of SNP marker polymorphism, mean minor allele frequency (MAF), observed heterozygosity (Ho), and expected heterozygosity (He), were calculated for both serologically positive and negative groups of sheep using PLINK 1.9. To compare the population structures and stratification across herds, principal component analysis (PCA) was applied to a genetic relationship–covariance matrix using GCTA (Genome-wide Complex Trait Analysis) 1.93.2 software (Yang et al., 2011). The results were further diagrammed as comprehensive two-dimensional scaling of the genetic variability within the analysed populations.

### 2.3 Assessment of the CLA infection status using ELISA

A commercial ELISA serological test (ELITEST CLA, HYPHEN BioMed, Neuville-sur-Oise, France) was used to detect any IgG antibodies against PLD in blood sera. This direct ELISA utilizes a recombinant form of the conserved *Cps* virulence factor PLD to detect anti-PLD IgG antibodies in sera from sheep and goats. Moreover, another commercially available ELISA ID Screen CLA Indirect assay (ID.vet Innovative Diagnostics, Grables, France) was used to confirm the *Cps* presence by detecting anti-PLD antibodies. All ELISA tests were performed as single determinations.

Both ELISAs were performed according to the manufacturer's instructions, and the resulting absorbance was measured at 450 nm using a Tecan microplate reader (Schoeller Instruments, Prague, Czech Republic). The cut-off values for the ELISAs were calculated as S/P % = (OD sample – OD negative control) / (OD positive control – OD negative control) × 100, where S/P is the sample-to-positive ratio and OD is the optical density, and were defined as follows: ≤ 40 % negative, 40 %–50 % questionable, and ≥ 50 % positive. The calculation of cut-off values and the interpretation of results followed the manufacturer's guidelines.

### 2.4 Phenotype categorization according to the serological status

Based on the results obtained from the ELISA tests, sheep on the four investigated farms with a proven occurrence of CLA were classified into two phenotypic groups: (1) healthy, uninfected animals without visible abscesses, consistently tested negative for the two independent ELISAs (ELITEST and ID Screen Indirect assay) in the observed years; and (2) diseased, CLA-affected animals, which were, on the contrary, always positive for both applied ELISA tests.

Sheep included in the study were tested for PLD Abs (antibodies) at least three times over a 4-year period, with annual testing conducted during the same period each year. Some animals had all four test results available, but a minimum number of three tests was required for inclusion in GWAS. To minimize the possibility of non-exposure bias, the negative animals were intentionally born in the same herd and during the same period as the positive animals.

The association study included a total of 321 adult female sheep (aged 2 to 8 years) of the purebred Suffolk breed, all with verified parentage, from four farms, comprising 143 positive and 178 negative ewes. Only female sheep were included in the GWAS because sufficient serological data were not available for male sheep. Based on data from farmers and their long-term experience, the typical live weight of adult Suffolk ewes under standard farming conditions is approximately 80 kg.

### 2.5 Genome-wide association study

A GWAS was conducted to assess the relationship between the sheep genome and the antibody response phenotype to the PLD antigen of *Cps*. Associations between the genotypes and the presence or absence of antibodies were analysed in a case–control study using genome-wide complex trait analysis with the “-mlma” option (mixed-linear-model-based association analysis) of GCTA 1.93.2 software (Yang et al., 2011). GCTA is a widely used software package for GWAS data analysis (Badia-Bringue et al., 2023; Hafliger et al., 2021), offering several advantages, including the ability to estimate the proportion of phenotypic variance explained by genome-wide SNPs and to correct for population structure and relatedness using a genomic relationship matrix (Yang et al., 2014). In contrast to single-SNP association analysis, GCTA fits the effects of all SNPs as random effects, estimating the total phenotypic variance captured by SNPs. Among the most well-known limitations of GCTA are that it primarily models additive genetic effects, potentially underestimating heritability by excluding non-additive interactions such as epistasis or dominance, and that its accuracy depends on the sample size, as well as SNP quality and density (Zhou, 2017).

The mixed linear model (MLM) can be represented by the following equation: y=a+bx+g+e . Here, y represents the phenotype; a represents the mean term; b represents the additive effect (fixed effect) of the SNP being tested for association; x represents the SNP genotype indicator variable, coded as 1 or 2; g represents the polygenic effect (random effect), assumed to be distributed as $N∼(0\sigma_{G}^{2})$ ; and e is the residual, assumed to be distributed as $N∼(0\;\sigma_{e}^{2})$ . The polygenic effect is the SNP cumulative effect; it is captured using the dense genomic relationship matrix (GRM), which is calculated using all SNPs. This allowed us to correct for population or relatedness structure in the data (Yang et al., 2014).

Because of a clear genetic differentiation among animals originating from separate heads (as shown by PCA analysis) and the increasing chance to contact with pathogen together with age, herd and age were included as covariates in the analysis. Benjamini–Hochberg correction with a 5 % genome-wide false discovery rate (FDR) was used to control for multiple testing. For the GWAS, single-marker p values were used to generate Manhattan and quantile–quantile (QQ) plots using the respective “manhattan” and “qq” functions implemented in the “qqman” R package (version 4.1.2).

### 2.6 Identification of SNPs and overlapping genes

The GGP 50K Ovine chip's design is based on the Ovis_Aries_1.0 and Oar_v3.1 reference assembly; therefore, the coordinates of the GWAS-identified SNPs were converted to ARS-UI_Ramb_v2.0 using LiftOver software (https://genome.ucsc.edu, last access: 17 October 2023) and the NCBI Genome Remapping Service (https://www.ncbi.nlm.nih.gov/genome/tools, last access: 17 October 2023, Kent et al., 2002). The identified SNPs' genomic localization and biological consequence of overlapping genes were determined using the Ensembl Variant Effect Predictor (VEP): https://www.ensembl.org/info/docs/tools/vep/index.html, last access: 8 April 2024. All of the identified functions of the genes and proteins were extensively searched using the GeneRIF tool in the NCBI Gene database (https://www.ncbi.nlm.nih.gov/gene/, last access: 17 April 2024) and UniProt Knowledgebase (https://www.uniprot.org/, last access: 17 April 2024).

### 2.7 Gene ontology annotation (GOA)

To investigate the functions of the underlying coding genes, we conducted functional enrichment analysis using an overrepresentation test (Khatri et al., 2012) based on the Gene Ontology database (https://geneontology.org/, last access: 29 April 2024) and the Kyoto Encyclopedia of Genes and Genomes (KEGG) (https://www.kegg.jp/, last access: 3 May 2024) implemented in the WEB-based GEne SeT AnaLysis Toolkit, WebGestalt online tool (Liao et al., 2019; Kanehisa and Goto, 2000).

GOA was conducted on genes located within ± 500 kb regions around associated SNP loci identified by the GWAS analysis. These regions were selected based on the hypothesis that SNPs in close proximity might be in linkage disequilibrium (LD) with causal variants influencing the trait of interest. The focus on ± 500 kb regions reflects the genomic scale at which LD is typically observed in sheep populations and the practical limits of the 50 K SNP chip design (Kijas et al., 2014).

Given the limited functional data available for *Ovis aries*, human and cattle genomes were used as references for GOA. Default parameters for the enrichment analysis were applied: minimum and maximum number of gene IDs in the ontological categories were 5 and 2000, respectively; the number of categories expected from set cover was 10, and the number of categories visualized in the resulting report was 40. The thresholds for the enriched gene ontology (GO) terms and pathways were set by applying the Benjamini–Hochberg correction to achieve a $p_{\mathrm{adj}}$ (FDR) value of 0.05. Redundancy reduction was achieved via the weighted set cover approach, which finds the top gene sets while maximizing gene coverage.

In addition, functional interactions between proteins encoded by some of the identified genes were investigated using STRING 12.0, a database of functional protein association networks (http://string-db.org/, last access: 20 November 2024) (Szklarczyk et al., 2023). STRING is specifically designed to identify known and predicted protein–protein interactions.

## 3 Results

### 3.1 Prevalence of antibodies against *Cps * in sheep farms

During the first serological screening of 30 monitored farms, it was found that, on average, 18.9 % of the animals (both sexes) were affected with CLA across 14 farms. The number of tested sheep and the average share of seropositive animals varied among the herds of the four investigated farms (B, D, L, and S) included in the GWAS:

- Herd B had approximately 370 sheep, with 22 % being seropositive on average over 4 years, corresponding to about 83 positive animals.
- Herd D comprised about 200 sheep, of which 25 % were seropositive on average, equating to approximately 49 positive animals.
- Herd L included around 150 sheep, with an average of 30 % being seropositive, representing about 45 positive animals.
- Herd S consisted of approximately 265 sheep, with 29 % being seropositive on average, corresponding to around 78 positive animals.

In the cohort of sheep included in the GWAS, ELISA results consistently indicated negative results for 178 out of 321 ewes over the 4 years, despite their exposure to CLA infection. These animals likely exhibited lower susceptibility or greater tolerance to *Cps* infection. Conversely, 143 ewes consistently tested positive in all serological tests, indicating that they might be more sensitive to infection.

To further explore sheep–pathogen interactions, the associations between the ovine genome and the production of CLA antibodies were investigated using the genotypes from the infected sheep with positive ELISA tests and the genotypes from the healthy sheep with negative ELISA tests.

### 3.2 Characterization of the polymorphism level and allelic distribution

Fundamental indicators of SNP polymorphisms within each of the tested sheep populations differing in their serological status were assessed by estimating the average share of SNP markers with an MAF ≥ 0.05 and the MAF and heterozygosity values. The results are presented in Table 1, with Fig. S1 and Fig. S2 provided in the Supplement. Genotyped SNPs overlapped with a total of 7511 coding genes; the average MAF was between 0.309 and 0.311. The proportion of SNPs with an MAF ≥ 0.05 was relatively high, accounting for more than 87.0 %, suggesting a considerable level of SNP polymorphism. The genetic variability parameters were comparable between healthy and diseased sheep, and the MAF distribution was well balanced; we did not observe substantial differences (Fig. S1). The results also revealed that the SNPs on chromosomes 19 and 23 (*OAR19* and *OAR23*, respectively) exhibited the highest MAF values in the Suffolk breed, while *OAR14* and *OAR18* had the lowest (Fig. S2).

**Table 1:** Comparison of basic descriptive statistics (mean values) for SNP genetic variability between healthy and diseased sheep.

Population	N	Ho	He	MAF	% of SNPs with	% of SNPs not	SNPs Δ Ho -
					MAF ≥ 0.05	in HWE	He ( p<0.05 )^*^
Healthy sheep	178	0.401 ± 0.112	0.398 ± 0.106	0.311 ± 0.122	87.41	1.14	2470
Diseased sheep	143	0.405 ± 0.115	0.397 ± 0.107	0.309 ± 0.123	87.11	1.14	2147

The abbreviations used in the table are as follows: N – number of tested sheep; Ho – observed heterozygosity; He – expected heterozygosity; MAF – minor allele frequency; HWE – Hardy–Weinberg equilibrium; “^*^” – number of SNPs with a significant difference between the observed and expected heterozygosity ( $\chi^{2}$ test).

To fully utilize the available SNP genotype information and explore the relationship between CLA and the sheep genome, we applied an approach based on identifying differences between the mean SNP relative allelic frequencies of “sensitive” (serologically positive) and “resistant” (long-term serologically negative) sheep. To be consistent, we compared the relative frequency of the identical alleles of each evaluated SNP. This approach further enabled the classification of SNPs according to their frequency difference among negative and positive animals. Most of the Ovine-chip-evaluated SNPs fell into the range from negligible (up to 0.02) to minor frequency differences (0.021–0.060), and only 716 SNPs (1.8 % of the evaluated SNPs) exceeded the 0.10 threshold. The results are displayed in Fig. S3, which is provided in the Supplement.

### 3.3 Genetic structure of sheep populations

**Figure 1:**
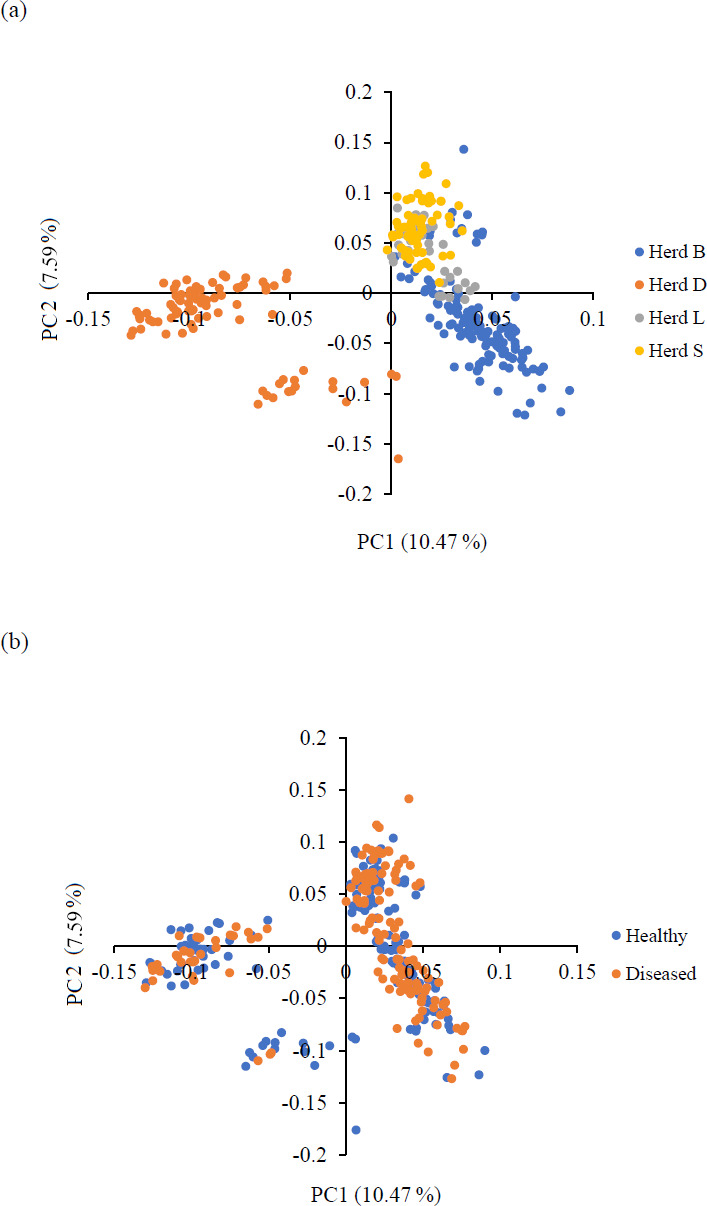
Principal component analysis (PCA) results. This is a picture of two-dimensional plots displaying the first two principal components (PCs) obtained from the PCA on the genomic relationships between the investigated sheep. **(a)** Genetic structure of the four examined sheep herds from farms affected with CLA. **(b)** Genetic structure of the investigated healthy (serologically negative) and diseased (serologically positive) sheep populations from the four genotyped herds.

Principal component analysis (PCA) was performed to assess the population structure and stratification, allowing visualization of the genetic background of the investigated sheep. PCA revealed the formation of three clusters: one with samples originating from three investigated herds and two with samples originating from one herd. In the PCA plot, the first principal component (PC), accounting for most of the variation in the animals' relationships (10.47 %), exhibited the highest discriminatory power, successfully separating the sheep of herd D from herds B, L, and S. The second PC, which accounted for 7.59 % of the variation in the animals' relationships, classified herd D into two groups. The healthy and diseased animals were evenly distributed among all clusters, enabling GWAS analysis. The PCA results are depicted in Fig. 1.

### 3.4 Genome-wide association study

In the current study, the associations between genotypes and CLA tolerance were investigated using the genotypes from the infected sheep with positive ELISA results and with the genotypes from the healthy sheep with negative ELISA results as a control population. The GWAS enabled a detailed investigation of the association between the genomic SNPs present in the ovine microarray and the occurrence of *Cps* antibodies in sheep over an extended period.

An overview of SNP markers indicative of CLA resistance or susceptibility is provided in the Supplement (Table S1). This subset consists of 686 SNPs with raw p values <0.05 in the GWAS analysis, overlapping 415 ovine genes annotated in the Ensembl gene database (Ensembl 112 gene set release). The majority of these SNPs exhibited notable differences in allelic frequencies (ranging from 5 % to 17 %) between serologically positive and negative sheep. Functional consequences of the indicative SNPs were evaluated using the Ensembl VEP tool (http://www.ensembl.org/index.html, last access: 8 April 2024). The analysis revealed that approximately 84.0 % of the SNPs were located within genes or in close proximity (up to 5000 bp), predominantly in introns and downstream variants. Among the coding sequences, missense variants accounted for 83 %, while synonymous variants constituted 17 % (Fig. S4 in the Supplement).

**Figure 2:**
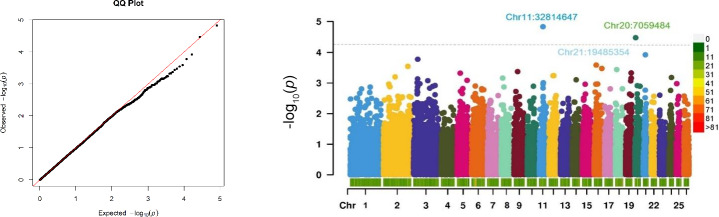
A QQ plot of the observed - log_10_ ( p values) versus expected values and a Manhattan plot of the GWAS for CLA antibodies. The Manhattan plot shows the - log_10_ ( p values) for the association between each SNP with the phenotype. The case–control GWAS was conducted on 321 Suffolk sheep with two phenotypes: cases with positive ELISA results ( N=143 ) and controls with negative ELISA results ( N=178 ). The dashed line indicates the threshold for suggestive genome-wide significance ( $p<4.42\times 10^{-5}$ ). The colour scale represents the density of SNP markers per 1 Mb of the genome. Chr denotes chromosome.

The relationship between SNP genotypes and the antibody response, which may indicate genetically driven susceptibility to CLA, is visualized in a Manhattan plot (Fig. 2). The QQ plot (Fig. 2) demonstrates that the observed p values closely align with the expected distribution under the null hypothesis, suggesting a well-calibrated GWAS. A slight upward deviation in the upper tail highlights the presence of potentially significant SNP associations with CLA antibody titres.

No SNPs reached Benjamini–Hochberg genome-wide significance. However, two SNPs (Chr11:32608530 and Chr20:7010930) with suggestive significance were identified, associated with the antibody response to *Cps* on chromosomes 11 and 20, with p values of less than 4.42 × 10^−5^. These suggestive SNPs map to chromosomal coordinates 32814646 for *OAR11* and 7059483 for *OAR20* based on the ARS-UI_Ramb_v2.0 genome assembly. The suggestive significance threshold, proposed by Lander and Kruglyak (1995), indicates one expected false positive per genome scan under the null hypothesis.

The most significant suggestive SNP, rs41700265, on chromosome 11, was located in the first exon of the *TRIM16* (tripartite-motif-containing 16) gene. This SNP represents a missense, potentially deleterious variant (proline/leucine amino acid exchange) likely to affect protein function, as predicted by the SIFT tool (https://sift.bii.a-star.edu.sg/, last access: 8 April 2024). The second SNP, located in an intergenic region on chromosome 20, was 9974 bp from the nearest adjacent gene, ENSOARG00020030814, a non-coding lncRNA involved in transcriptional regulation.

BioMart (Ensembl) was used to investigate genomic regions spanning ± 500 kb around the two suggestive SNPs. Analysis identified 19 genes near the SNP on *OAR11* and 18 genes near the SNP on *OAR20*, several of which may have functional relevance to CLA susceptibility. The findings are summarized in Table 2.

**Table 2:** Genes identified within ± 500 kb regions around the two suggestively associated SNPs, with the genomic location, p values, amino acid changes, and predicted functional consequences of the SNPs.

SNPs	Existing	OAR: bp^*^	Alleles	p value	Consequence	AA	Genes within region
	variation						
Chr11:32608530	rs417000265	11: 32814646	G/A	$1.49\times 10^{-5}$	Missense variant	P/L	*PMP22, TEKT3, CDRT4, TRIM16, ZNF286A, ZNF624, ZNF287, LRRC75A, SNORD65, SNORD49A, SNORD49B, TRPV2, UBB, PIGL, NCOR1, TTC19, ZSWIM7, ADORA2B, SPECC1*
Chr20:7010930		20: 7059483	T/C	$3.41\times 10^{-5}$	Intergenic variant		*MLIP, LRRC1, KLHL31, U6, GCLC, HLA-DOB, TAP2, PSMB8, TAP1, HLA-DMB, HLA-DMA, BRD2, HLA-DOA, COL11A2, RXRB, SLC39A7, HSD17B8, RING1*

^*^ Base pair positions are based on ovine reference ARS-UI_Ramb_v2.0. The abbreviations used in the table are as follows: SNP – single-nucleotide polymorphism; OAR – ovine chromosome; bp – base pair positions; AA – amino acid change.

### 3.5 Gene ontology annotation

The gene ontology analysis was conducted to explore the functional roles of genes identified within the genomic regions surrounding the two candidate SNPs. Specifically, we focused on genes located within ± 500 kb of the SNPs on *OAR11* and *OAR20*, which showed suggestive associations with the production of specific Abs against *Cps*. This approach resulted in a pool of 37 proximal genes: 19 genes on *OAR11* and 18 genes on *OAR20*. The symbols of these genes are listed in Table 2. These genes were further analysed using GO tools to identify enriched biological processes, molecular functions, cellular components, and pathways that might provide insights into their roles in CLA susceptibility. Among them, 31 were annotated to functional categories listed in the genome reference used for the enrichment analysis implemented in WebGestalt tools. The most representative gene sets covering these ontological categories are shown in Table 3.

**Table 3:** Enrichment of gene ontology terms and pathways related to the CLA antibody response based on FDR values. Gene ontology analysis was performed on genes located within ± 500 kb of the SNPs on *OAR11* and *OAR20*, which showed suggestive association with the production of antibodies against *Cps*. The significant ontological categories are presented.

Gene set	Description	Size	Overlap	Enrichment ratio	p value	FDR
Biological process						
GO:0019882	Antigen processing and presentation	116	6	29.96	$4.0\times 10^{-8}$	0.00036
GO:0002478	Antigen processing and presentation of exogenous peptide antigen	40	4	57.92	$6.3\times 10^{-7}$	0.00189
Cellular component						
GO:0042613	MHC class-II protein complex	16	3	107.42	$2.7\times 10^{-6}$	0.003
GO:0042824	MHC class-I peptide-loading complex	8	2	143.23	$8.2\times 10^{-5}$	0.03
KEGG pathway						
hsa04145	Phagosome	152	6	17.06	$8.5\times 10^{-7}$	0.0001
hsa04659	Th17 cell differentiation	108	5	20.01	$3.7\times 10^{-6}$	0.0002
hsa04672	Intestinal immune network for IgA production	49	4	35.28	$4.1\times 10^{-6}$	0.0002
hsa05168	Herpes simplex virus 1 infection	512	8	6.75	$9.6\times 10^{-6}$	0.0003

**Figure 3:**
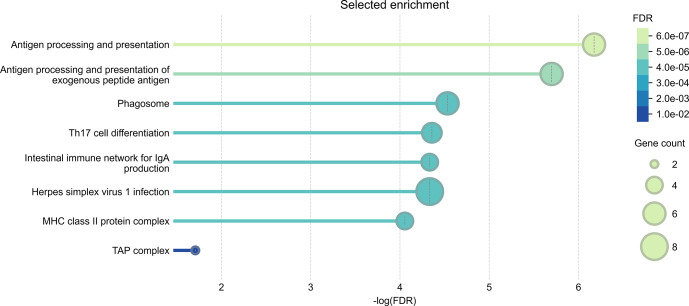
Selected enrichment of biological processes, cellular components, and KEGG pathways, prioritized by the highest FDR values and reduced redundancy through the weighted set cover approach. Analysis was conducted for genes located within ± 500 kb of two suggestive SNPs on *OAR11* and *OAR20*, identified in Suffolk sheep through GWAS of *Cps* antibody seroprevalence, using WebGestalt and the STRING database with default settings.

The results of the gene ontology analysis indicated statistically significant enrichment of genes involved in antigen processing and presentation (GO:0019882), particularly the processing and presentation of exogenous peptide antigens (GO:0002478). This suggests that key pathways related to immune surveillance and antigen recognition can be active in the response to CLA. Cellular component analysis further revealed enrichment in the major histocompatibility complex (MHC) class-II protein complex (GO:0042613) and the MHC class-I peptide-loading complex (GO:0042824), also known as the TAP complex, indicating the involvement of both class-I and class-II antigen presentation mechanisms. KEGG pathway analysis identified additional immune-related pathways as significantly enriched. These include the Phagosome pathway (hsa04145), which is critical for antigen uptake and processing, and the Th17 cell differentiation pathway (hsa04659), reflecting the potential role of Th17 cells in regulating inflammation and immune defence. The intestinal immune network for IgA production (hsa04672) pathway indicates the involvement of mucosal immunity. The Herpes simplex virus 1 infection pathway (hsa05168) may point to broader antiviral defence mechanisms, reflecting the known similarity between the immune evasion strategies of viruses and those employed by *Cps * (Oliveira Neto et al., 2017). These findings were further supported by the protein–protein interaction (PPI) network analysis performed in STRING. The STRING database aims to collect, score, and integrate all known and predicted protein–protein association data and enables functional protein association network analysis and visualization (Szklarczyk et al., 2023). The results of the functional enrichment analysis are shown in Fig. 3.

**Figure 4:**
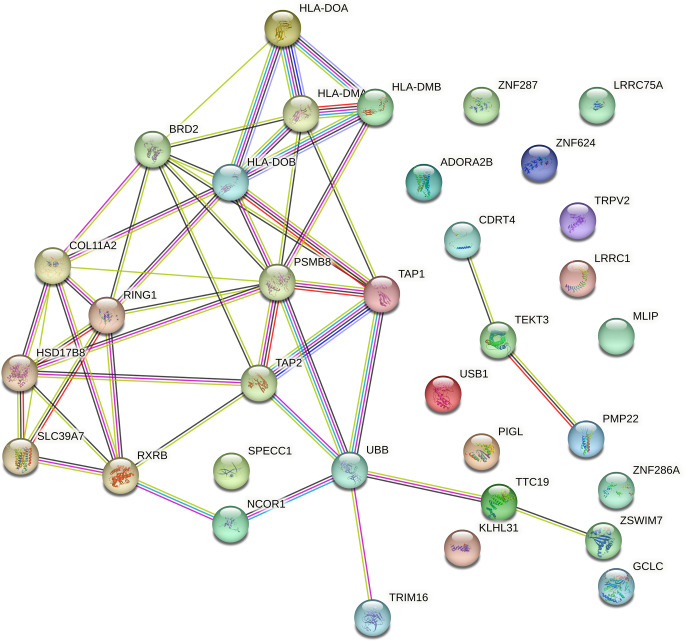
Protein–protein interaction network of proximal genes located within ± 500 kb of two suggestive SNPs on *OAR11* and *OAR20*, identified in Suffolk sheep through GWAS of *Cps* antibody seroprevalence, using the STRING database. Interactions include both physical and functional associations derived from various sources: genomic context, high-throughput experiments, conserved co-expression, and prior knowledge. The network highlights a PSMB8-centred subnetwork and interactions involving TRIM16, with most proteins showing functional relationships. Data are based on STRING medium confidence scores ( ≥ 0.4). Each node represents a protein, and each edge indicates an interaction.

The PPI network was constructed based on several evidence channels, including genomic neighbourhood, gene fusions, co-occurrence across genomes, co-expression, experimental and biochemical data, curated database associations, and co-mentions in PubMed abstracts (Alonso-Hearn et al., 2019). Only functional interactions with a medium confidence score ( ≥ 0.4) were included, resulting in a network that highlights biologically meaningful connections. This analysis revealed that proteins encoded by the proximal genes exhibited significantly more interactions than expected for a random set of proteins (number of nodes = 34; observed number of edges = 49; expected number of edges = 5; PPI enrichment p value $<\;1.0\times 10^{-16}$ ; Fig. 4), suggesting that these genes which are situated on the two different ovine chromosomes are functionally interconnected.

## 4 Discussion

This study represents the first attempt to investigate the genetic associations related to the antibody response against *Cps *in ordinary flocks of sheep. Two commercial diagnostic ELISA kits were used to detect IgG antibodies against PLD, a recognized indicator of CLA in sheep. PLD is not known to be produced by other sheep pathogenic bacteria, making it a very specific test. The ELITEST CLA is a widely accepted test because of its cost-efficiency and acceptable sensitivity (87 %) and specificity (98 %) (Gascoigne et al., 2020). This methodology aligns with established practices validated for their effectiveness and reliability in CLA diagnostics in sheep populations (Nassar et al., 2014; Alves et al., 2020). However, limitations of ELISA tests have been noted, particularly their reduced specificity and sensitivity in detecting subclinical CLA cases in small ruminants (Costa et al., 2020). Furthermore, serological tests might not be able to distinguish animals that have cleared infections from those with active lesions (Gascoigne et al., 2020). Currently, there is limited knowledge about the persistence of specific immunoglobulins in ovine serum and their production during the course of the disease in commercial flocks. Nevertheless, extensive research has examined antibody responses in animals experimentally infected with *Cps* PLD, aiming to aid in effective vaccine development (Dominguez et al., 2021). An experimental study assessing the kinetics of IgG production in goats, conducted by Paule et al. (2003), demonstrated maximum antibody titres within 11–21 d following primary infection. However, not all animals in that study remained seropositive throughout the 20-week follow-up, suggesting transient humoral immunity (Paule et al., 2003).

Conversely, our findings indicate longer-lasting Abs in naturally infected sheep from large flocks. This could be attributed to repeated antigenic stimulation in chronically infected animals, where *Cps* intermittently re-enters the lymphatic and blood vessels after macrophage rupture (Bastos et al., 2012). Reinfection from other flock members might also contribute to prolonged antibody presence, although the relative importance of these mechanisms in commercial flocks remains unclear. The presence of specific immunoglobulins at a given time point likely reflects recent antigen exposure and immune response dynamics, rather than a direct link to CLA genetic susceptibility.

These antibodies may predominantly arise from short-lived plasma cells, which are responsible for immediate antibody production upon antigen exposure. In contrast, long-lived memory B cells play an essential role in ensuring rapid and robust antibody responses upon re-exposure to antigens. Recent transcriptomic and proteomic studies in CLA-affected small ruminants have reported increased expression of the tumour necrosis factor (TNF) receptor superfamily member ^13^C, which is critical for mature B-cell survival. Additionally, upregulated genes associated with the B-cell receptor-signalling pathway and adaptive somatic recombination of immunoglobulin domains suggest that infected animals are capable of developing antigen-specific immune memory (Fu et al., 2022; Kyselova et al., 2023).

Unfortunately, in large-scale commercial flocks, detecting spontaneous recovery or identifying animals with genetic resistance to CLA is highly challenging. Nevertheless, healthy, serologically negative ewes exposed long-term to the pathogen may have developed more efficient biological, metabolic, or innate immune mechanisms to inhibit bacterial spread.

GWASs are capable of identifying genetic variants associated with diseases across the entire genome rather than just a single gene or region. However, the significance of individual SNPs in GWASs can be heavily influenced by the size and diversity of the population sample. This can make detecting markers with low influence more challenging, and SNPs of lower significance may go undetected (Kravitz et al., 2021). In this study, the limited sheep population likely contributed to the reduced statistical power to identify strongly significant SNPs (Mkize et al., 2021). Despite identifying a few outliers in one of the investigated herds through PCA, no animals were excluded due to sample size constraints. Instead, the focus was on achieving a balanced distribution between positive and negative ewes within the clusters, which was deemed more critical. Additionally, the applied statistical model corrected for population substructures (Yang et al., 2014), which is an additional argument for keeping all sheep in the analysis. We applied a constant set of criteria, including regular repeated testing and the intentional selection of negative animals from the same herd and period as positive animals, to ensure the reliability of our findings.

Although no SNPs reached genome-wide significance, two suggestive SNPs were identified on chromosomes 11 and 20. Notably, one potentially deleterious SNP was located in the first exon of the *TRIM16* gene, which has been implicated in innate immunity and infectious disease responses (Bell et al., 2012; Fernandes et al., 2023). Recent research underscores the importance of TRIM21 in mice for controlling *Cps* infections by limiting bacterial replication and regulating proinflammatory cytokine secretion (Tan et al., 2021). Additionally, elevated transcription of TRIM45 was noted in CLA-exposed sheep blood transcriptomes (Kyselova et al., 2023).

TRIM16, an E3 ubiquitin ligase, plays a critical role in immune regulation by enabling interleukin-1 beta (IL-1 β ) production, a key proinflammatory cytokine in acute immune responses and bacterial defence (Vijayaraj et al., 2021). In macrophages, TRIM16 is essential for selective autophagy, ubiquitination, and repair of lysosomal and phagosomal damage (https://www.uniprot.org/uniprotkb/O95361/entry, last access: 17 April 2024). Autophagy serves as a vital innate immune mechanism, targeting intracellular bacteria, damaged vacuoles, and phagosomes to restrict bacterial growth (Huang and Brumell, 2014). The missense *TRIM16* variant rs41700265 identified in this study may influence CLA susceptibility by modulating autophagic responses. Moreover, TRIM16 coordinates the recognition of membrane damage with core autophagy regulators (ATG) and protects cells from *Mycobacterium tuberculosis* invasion (Chauhan et al., 2016). STRING network analysis highlighted TRIM16's involvement in antigen presentation processes via the MHC, bridging innate and adaptive immunity. These findings emphasize the dual role of TRIM16 in autophagy and antigen recognition, critical for defence against *Cps* (Bastos et al., 2012).

The GOA focused on genes located within ± 500 kb of the two indicative SNPs, enabling the identification of enriched biological processes and pathways potentially associated with CLA. This approach is particularly useful in complex polygenic traits like CLA, where multiple genes contribute to the phenotype. Similar methodologies have been successfully applied in livestock studies to explore the genetic basis of complex traits, particularly those involving immune responses (Otto et al., 2018; Raeesi et al., 2017). In our analysis, several genes in these regions, including those contributing to the formation of the MHC class-II complex, exhibited strong functional interactions in STRING analyses, underscoring their importance in immune processes such as antigen processing and presentation. The region on *OAR20* is also functionally interconnected with *TRIM16*, an important regulator of autophagy and IL-1 β production. These pathways are vital for the adaptive immune response to bacterial infections, including *Cps*.

Although applying case–control GWAS to such traits presents challenges due to polygenic inheritance and the involvement of numerous loci, it remains a valuable method for uncovering marker variants in proximity to causal variants. For instance, genes such as *TAP1 * (antigen peptide transporter 1), *TAP2* (antigen peptide transporter 2), *PSMB8* (proteasome subunit beta type-8), and those encoding components of the MHC class-II protein complex: *HLA-DOA* (HLA class-II histocompatibility antigen, DO alpha chain), *HLA-DMA* (HLA class-II histocompatibility antigen, DM alpha chain), and *HLA-DOB* (HLA class-II histocompatibility antigen, DO beta chain), participate in pathways essential for immune surveillance. The TAP complex, involved in the transport of antigenic peptides to MHC class-I molecules, plays a dual role in modulating both class-I and class-II antigen presentation, indicating its broader significance in adaptive immunity (Basler et al., 2013; Neefjes et al., 2011). These findings align with previous studies on ruminant immunity, which highlight the centrality of antigen processing in resistance to bacterial pathogens including *Cps* (Frie et al., 2017; Fu et al., 2021; Garcia et al., 2023; Gutiérrez et al., 2017).

In sheep, several studies have been conducted on the involvement of MHC genes and antigens in genetic resistance to diseases, the majority being concerned with gastrointestinal nematodes and bacterial infections (Dukkipati et al., 2006; Gossner et al., 2017; Purdie et al., 2019). Recent findings based on long-term antibody measurements and SNP genotyping have further highlighted the role of MHC class-II genes in resistance. Evidence from ovine chromosome 20 suggests their potential as targets for selective breeding to limit infections such as paratuberculosis (Usai et al., 2024). Interestingly, another line of research has focused on predicting MHC class-I and class-II epitopes from target proteins of *Cps* to develop effective vaccines that elicit protective and long-lasting immune responses against CLA (Droppa-Almeida et al., 2018).

Additionally, KEGG analysis identified the phagosome and Th17 cell differentiation pathways as significantly enriched in the investigated pool of proximal genes. The phagosome pathway is a cornerstone of innate immunity, mediating antigen uptake, processing, and presentation. Phagosomes not only enable the clearance of pathogens but also provide a platform for the cross-talk between innate and adaptive immunity. Similarly, the Th17 cell differentiation pathway highlights the potential of Th17 cells in the immune response to *Cps*, particularly through the production of interleukins IL17A and IL22, which stimulate epithelial and inflammatory responses (Korn et al., 2009; DeKuiper and Coussens, 2019). These cytokines were broadly discussed in a recent article (Valeri and Raffatellu, 2016), as they contribute to the clearance of the pathogen and help control its dissemination, linking mucosal immunity with humoral responses. This underscores the need for effective Th17 activity to control the spread of infection both on mucosal surfaces and within the organism (Bastos et al., 2012).

The intestinal immune network for IgA production, another enriched pathway, reflects the importance of mucosal immunity in the context of bacterial infections. Mucosal immunity, mediated by IgA, may play a role in reducing the bacterial load at the initial site of infection, thus mitigating the systemic spread and chronic abscess formation characteristic of CLA. The identification of this pathway alongside others related to antigen processing suggests a multifaceted immune response involving both systemic and localized immunity (Chase and Kaushik, 2019; Pabst, 2012).

Interestingly, the enrichment of the Herpes simplex virus 1 infection pathway might indicate broader immune evasion strategies utilized by *Cps*. This bacterium shares similarities with viral pathogens in its ability to evade host defences, including mechanisms that interfere with antigen processing and presentation (Oliveira Neto et al., 2017). The overlap between these pathways further supports the hypothesis that immune system pathways with general relevance to pathogen recognition and clearance are critical in determining CLA susceptibility.

These findings emphasize the utility of GWAS as a tool for exploring complex traits and their associated genetic architectures, paving the way for future functional studies and breeding strategies aimed at improving disease resistance in sheep. However, while enhancing immunocompetence through selective breeding holds promise for increasing resistance to CLA, it is essential to consider potential trade-offs. For instance, stronger immune responses may lead to increased energy demands or risks of immunopathology, such as excessive inflammation or autoimmunity. Moreover, theoretical models suggest that trade-offs between tolerance and resistance may arise when these traits are governed by distinct immune pathways competing for energy resources (Knap and Doeschl-Wilson, 2020). These trade-offs, which may involve increased energy demands or risks of immunopathology (e.g. inflammation or autoimmunity), could influence the overall fitness and productivity of sheep in commercial flocks.

The integration of genomic and phenotypic data can help balance these aspects by identifying genetic markers that optimize both disease resistance and other economically important traits, such as growth and reproduction. However, even if unfavourable associations exist, breeders can create selection indices that include traits with unfavourable associations and maximize the desired responses while attempting to minimize undesirable effects (Brito et al., 2021; Stear et al., 2001). Further research is needed to quantify these trade-offs under diverse environmental and management conditions to ensure that breeding for resistance does not inadvertently compromise overall flock performance or other economically important traits.

It is important to note that our study, while providing valuable initial insights, should be considered preliminary. A more extensive and genetically diverse sample population could help validate the findings and ensure that they are broadly applicable across different breeds and environmental conditions. Future research should apply more frequent measurements of antibody levels and other methods for the diagnosis and validation of the results. Furthermore, incorporating animal pedigrees into the GWAS can significantly enhance the study's ability to detect genuine genetic associations with diseases or traits by providing a clearer picture of the genetic landscape and its influence on the studied outcomes. These improvements will lead to more reliable and actionable findings informing breeding programmes and disease management strategies.

## 5 Conclusions

Our pilot study on *C. pseudotuberculosis* infection in Suffolk sheep has yielded critical insights into the serological, genetic, and functional aspects of CLA. The application of ELISA tests for antibody detection against the PLD antigen has been effective in differentiating between CLA-affected and healthy sheep. This approach aligns with established serological methods, reinforcing the role of ELISA in accurate disease diagnosis.

The genome-wide association study conducted as part of our research revealed suggestive genetic markers linked to CLA, particularly highlighting the significance of SNPs on chromosomes 11 and 20. Notably, *TRIM16* was identified as a key gene of interest, with its role in autophagy and immune regulation linking it to sheep resistance to CLA. Functional enrichment analyses further highlighted immune-related pathways, including antigen processing and presentation, mucosal immunity through IgA, and Th17 cell differentiation, demonstrating the multifaceted nature of the immune response to CLA.

These findings represent a further step in understanding the genetic and immunological mechanisms underlying CLA and provide a foundation for developing targeted interventions and selective-breeding strategies. Future research should expand on these results by incorporating genetically diverse populations, longitudinal antibody measurements, and multi-omics approaches to validate and refine genetic markers and pathways. Overall, this work contributes to veterinary medicine and animal genetics, offering novel insights for the effective management and control of CLA in sheep.

## Supplement

10.5194/aab-68-109-2025-supplement The supplement related to this article is available online at https://doi.org/10.5194/aab-68-109-2025-supplement.

## Data Availability

The research data from the study are available from the corresponding author upon reasonable request for interested members of the scientific community.
