# Supplementary material for: Genome-wide association study of the antibody response to Corynebacterium pseudotuberculosis in sheep
Source: Arch Anim Breed. 2025 Feb 11;68(1):109–24. doi: 10.5194/aab-68-109-2025 (PMC13239483; doi:10.5194/aab-68-109-2025)
Supplement: The supplement related to this article is available online at https://doi.org/10.5194/aab-68-109-2025-supplement. [file aab-68-109-2025-supplement.zip › Supplementary material Fig.S1-Fig.S4.docx]

**Supplementary material**

**Figure S1-S4**

**Characterization of the polymorphism level and allelic distribution**

Figure S1: The number of SNPs with the average MAF (MAF allelic distribution) estimated for all sheep included in the GWAS study. All SNPs that passed the genotype quality control were considered. MAF, minor allelic frequency.

Figure S2: The average MAF of SNPs evaluated per ovine autosome in the sheep included in the GWAS study. All SNPs that passed the genotype quality control were considered. MAF, minor allelic frequency. OAR, *Ovis aries* chromosomes.

Figure S3: Graph of differences in SNP allelic frequencies between serologically negative (healthy) and positive (diseased) sheep involved in the GWAS study. Each bar of the plot represents the SNP number within the evaluated frequency range. SNP, single nucleotide polymorphism.

**Genome-wide association study**

Figure S4: Distribution of the gene consequences of all SNPs putatively associated (*p*_raw_ value < 0.05) with CLA antibody response according to the Ensembl Variant Effect predictor (VEP).
