# Supplementary material for: Genome-wide association study of the antibody response to Corynebacterium pseudotuberculosis in sheep
Source: Arch Anim Breed. 2025 Feb 11;68(1):109–24. doi: 10.5194/aab-68-109-2025 (PMC13239483; doi:10.5194/aab-68-109-2025)
Supplement: The supplement related to this article is available online at https://doi.org/10.5194/aab-68-109-2025-supplement. [file aab-68-109-2025-supplement.zip › aab-68-109-2025-supplement-title-page.pdf]

Supplement of Arch. Anim. Breed., 68, 109–124, 2025
https://doi.org/10.5194/aab-68-109-2025-supplement
© Author(s) 2025. CC BY 4.0 License.

Open Access
Archives Animal Breeding

Supplement of

Genome-wide association study of the antibody response to *Corynebacterium pseudotuberculosis* in sheep

Jitka Kyselová et al.

Correspondence to: Jitka Kyselová (kyselova.jitka@vuzv.cz)

- aab-68-109-2025-supplement-title-page.pdf
- Supplementary material Fig.S1-Fig.S4.docx
- Supplementary table S1 fin.xlsx

The copyright of individual parts of the supplement might differ from the article licence.
